# Phase Transition Kinetics in Austempered Ductile Iron (ADI) with Regard to Mo Content

**DOI:** 10.3390/ma13225266

**Published:** 2020-11-21

**Authors:** Martin Landesberger, Robert Koos, Michael Hofmann, Xiaohu Li, Torben Boll, Winfried Petry, Wolfram Volk

**Affiliations:** 1 Chair of Metal Forming and Casting, Technical University of Munich, Walther-Meissner-Strasse 4, 85748 Garching, Germany; wolfram.volk@utg.de; 2 Heinz Maier-Leibnitz Zentrum (MLZ), Technical University of Munich, Lichtenbergstrasse 1, 85748 Garching, Germany; robert.koos@frm2.tum.de (R.K.); michael.hofmann@frm2.tum.de (M.H.); xiaohu.li@hzg.de (X.L.); winfried.petry@frm2.tum.de (W.P.); 3 Institut für Angewandte Materialien—Werkstoffkunde (IAM-WK), Karlsruhe Institute of Technology, Engelbert-Arnold-Strasse 4, 76131 Karlsruhe, Germany; torben.boll@kit.edu

**Keywords:** ADI, phase transition kinetics, retained austenite, molybdenum, atom probe tomography, neutron diffraction, dilatometry

## Abstract

The phase transformation to ausferrite during austempered ductile iron (ADI) heat treatment can be significantly influenced by the alloying element Mo. Utilizing neutron diffraction, the phase transformation from austenite to ausferrite was monitored in-situ during the heat treatment. In addition to the phase volume fractions, the carbon enrichment of retained austenite was investigated. The results from neutron diffraction were compared to the macroscopic length change from dilatometer measurements. They show that the dilatometer data are only of limited use for the investigation of ausferrite formation. However, they allow deriving the time of maximum carbon accumulation in the retained austenite. In addition, the transformation of austenite during ausferritization was investigated using metallographic methods. Finally, the distribution of the alloying elements in the vicinity of the austenite/ferrite interface zone was shown by atom probe tomography (APT) measurements. C and Mn were enriched within the interface, while Si concentration was reduced. The Mo concentration in ferrite, interface and austentite stayed at the same level. The delay of austenite decay during Stage II reaction caused by Mo was studied in detail at 400 °C for the initial material as well as for 0.25 mass % and 0.50 mass % Mo additions.

## 1. Introduction

Heat treatment of iron materials has a long history but is still a matter of intensive research due to its complexity [1,2,3,4]. Phase transformation, diffusion and segregation at different temperatures in dependence of alloying elements, initial state and time allow in the case of austempered ductile iron (ADI) a wide field of properties. Due to its ausferritic microstructure, tensile strengths of up to 1600 MPa can be achieved as well as elongation at fracture of up to 15% [5]. The material has various advantages for engineering applications, where high strength or wear resistance are important [6]. The microstructure consists of acicular ferrite, retained austenite, graphite nodules and carbides. Ni, Cu and Mo are known as the main alloying elements for influencing the ADI heat treatment and phase transition kinetics [7]. The amount and stability of the retained austenite, depending on the austenitization and austempering temperatures, change the characteristic of the TRIP effect appearing under plastic deformation or low temperature [8,9]. Strain induced martensite formation and phase transition kinetics within Ni alloyed austempered ductile irons were studied previously with neutron diffraction by Saal et al. [10]. Ni effectively shifts the formation of pearlite during quenching to a later onset and impedes the formation of ausferrite. The retained austenite content after austempering is elevated by this element, while the maximum carbon content is decreased. Cu is related to Ni as it also segregates negatively and impedes pearlite formation and stabilizes austenite. MgCu${}_{2}$ particles form in the vicinity of graphite nodules which are detrimental to fatigue properties in the presence of Mg and Cu contents above 0.8 mass % [11]. Being one of the central alloying elements [12], the role of Mo in ductile iron has been the topic of several studies since the discovery of ADI in the 1960s. Yazdani and Elliott [13,14,15,16] did a detailed study to clarify the influence of Mo on phase transition kinetics. They identified the process window ex-situ by means of hardness measurements, metallography and X-ray diffraction. The influence of the austenitization temperature on hardenability (full ausferritic matrix) was investigated as well as different Mo contents. The impeded reaction is explained by reaction speed differences between intercellular regions and the eutectic cells surrounding the graphite nodules. This is caused by segregation of Mo and Mn, which are both impeding the reaction from austenite to ausferrite. The effect can be so strong that the transformation to ausferrite in the intercellular regions has not started before the decomposition in austenite within the eutectic cells has begun. However, only a few further studies in the last years covered Mo as alloying element in ductile irons. Dekker et al. [17] conducted a longtime study on the formation of Mo carbides at the grain boundaries and within the austenite grains at elevated temperatures of up to 10k hours. They combined thermodynamic simulations with experimental procedures and found that a value of 0.27 mass % Mo is necessary for the precipitation of MoC within the austenite grains of the investigated ductile iron. Mixture carbides located at the austenite grain boundaries are metastable and can be dissolved at 550 °C. Gazda and Warmuzek [18] investigated the thermal stability of ausferrite with differential scanning calorimetry and dilatometry. They reheated ADI samples and discussed the occurring phase transitions in detail. Fundamental works on segregation were reviewed by Dorazil [19]. More recently, Domeij et al. [20] published wavelength dispersive spectroscopy measurements that show the present local material composition between the nodules. Mn and Mo are enriched in intercellular regions while earlier solidified locations close to the nodules contain more Si, Cu and Ni. Interface zones of austenite and ferrite are intensively investigated with atom probe tomography at bainitic structures within steels. Recently, carbide free bainitic steels have been developed utilizing Si to avoid cementite formation [21]. The composition of such steels is similar to the matrix of ADI materials, as the steels have a C content of 0.8 mass % in addition to Si contents of over 1 mass %. This class of material will be used as reference for discussing the presented atom probe tomography (APT) results in ADI.

**Figure 1:**
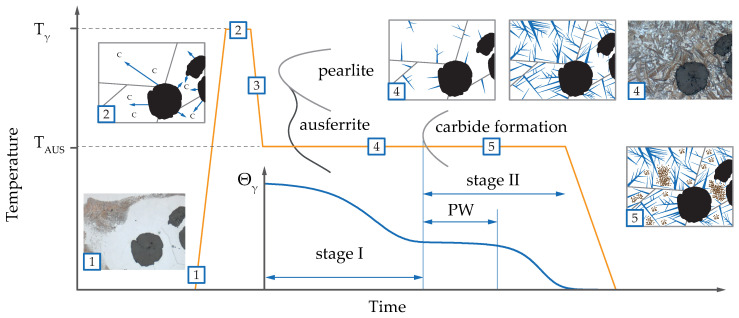
The course of an ADI heat treatment is exemplarily shown. Starting from the initial state at (1) over heating to austenitization temperature $T_{\gamma }$ (2), followed by quenching (3) to annealing at austempering temperature $T_{aus}$ and subsequent cooling to room temperature. The evolution of austenite phase fraction $\Phi_{\gamma }$ during austempering is schematically depicted. Ausferritic microstructure is formed in the Stage I reaction (4) and followed by the decomposition of retained austenite (5) into carbides and ferrite (Stage II). As long as the loss in austenite fraction is small, the process window (PW) for industrial ADI heat treatment is defined.

In this work, global in-situ and local ex-situ methods are combined to characterize the influence of Mo in detail. Transformation times and their effect on the phase transformation during quenching and isothermal heat treatment are investigated through neutron diffraction in combination with dilatometry. The distribution of Mo and interface composition between ferrite and austenite grains is shown with several APT measurements. Figure 1 shows the typical ADI heat treatment from initial state (1), austenitization (2), quenching (3) and finally to austempering (4). The latter divides into Stage I reaction, the process window which opens after achieving a full ausferritic microstructure, and the further decomposition of austenite into ferrite and carbides (Stage II reaction). The Stage II reaction is indicated by a loss in hardness and tensile strength and needs to be avoided during industrial heat treatment. Our in-situ studies follow the transformation of austenite and its enrichment with C directly. This allows us to evaluate the phase transition kinetics and therefore the process window start and end times with high accuracy. With the in-situ combination of dilatometry and neutron diffraction, the macroscopic length change can be directly evaluated in the context of C diffusion and phase transition. Further, atom probe tomography enabled localizing C, Mo, Mn, Si and Cr with a much higher level of detail than in previous studies.

## 2. Materials and Methods

### 2.1. Material Composition

A GJS 400-15 [22] nodular cast iron was used as base material (M1) for the alloying process with 0.25 mass % Mo (M2) and 0.50 mass % Mo (M3). The alloy compositions are listed in Table 1. The composition was measured by spark emission spectroscopy for all elements on white solidified specimens. The alloys were prepared in an industrial process at Bosch Rexroth AG (Lohr am Main, Germany) consisting of melting the constituents in an induction furnace with subsequent Mg treatment and then inoculated during casting. According to DIN EN ISO 945 [23], the nodule count was calculated to 158/mm${}^{2}$, 150/mm${}^{2}$ and 131/mm${}^{2}$ for M1–M3, respectively. The average nodule diameter was 34 μm (M1), 35 μm (M2) and 40 μm (M3). The average roundness value was 0.6. Particles smaller than 10 μm were not taken into account for nodule count, average diameter and roundness calculation.

### 2.2. Heat Treatment

The specimens (6 mm dia. × 18 mm in length) were heated in a mirror furnace with a rate of 3.3 °C/s to 900 °C by 4 halogen lamps with a power of 600 W in total within 0.5 bar Ar atmosphere. The use of Ar gas for quenching provides a cooling rate high enough avoiding the formation of pearlite. The critical linear cooling rate (to avoid pearlitic transformation start) for the base material is approximately 20 °C/s [19]. Within the mirror furnace, it took 25 s to overcome the temperature difference from 900 to 400 °C, resulting in an average cooling rate of 20 °C/s, which might have led to pearlite formation. It has to be considered that the cooling rate imposed by the Ar gas stream is not constant. During the interval from 800 to 500 °C, where pearlite formation is possible after crossing its critical temperature, the cooling rate is higher than 25 °C/s and reaches a maximum of 74 °C/s. Metallography proved the absence of pearlite in all investigated samples. Meier [24] described the exact experimental setup and further geometric details of the furnace that was used at the STRESS-SPEC instrument [25] for in-situ neutron diffraction studies. For the thermal treatment in the dilatometer experiments, heating and cooling rates equal to the mirror furnace experiments were chosen to achieve comparability.

### 2.3. Metallography and Interrupted Heat Treatment

Specimens were ground and polished subsequently at 6 μm, 3 μm and 1 μm steps, and 3% Nital served as etching agent. Micrographs were taken with an AxioCam MRc5 mounted on a Axioplan 2 microscope (Carl Zeiss AG, Oberkochen, Germany).

To follow the decomposition of ausferrite, interrupted heat treatment micrographs of the same specimen were taken at different times during austempering. Therefore, the isothermal heat treatment in the mirror furnace was interrupted, the specimen repolished at 1 μm step and etched at the top face. After taking micrographs the specimen was reheated to austempering temperatures (300–400 °C). The micrographs are taken at the same position and orientation of the sample. A loss of about 0.1 μm in height has to be considered.

### 2.4. Neutron Diffraction

The neutron diffraction experiments were carried out at the angle dispersive strain scanner STRESS-SPEC at the high-flux neutron source FRM II of the Heinz Maier–Leibnitz Zentrum (MLZ) [26,27]. Monochromatic neutrons with a wavelength of 0.21 nm were produced by a Ge(311) mosaic single-crystal monochromator [28] (flux on sample 2 × 10${}^{7}$ n cm${}^{-2}$ s${}^{-1}$). Neutron optical elements defined a gauge volume of 5 × 5 × 5 mm${}^{3}$ within the mirror furnace samples. A detector [29] with an active area of 256 × 256 mm${}^{2}$ (1 mm${}^{2}$ effective pixel size) captured the scattered neutrons at a distance of 1.035 m for an interval of 2Θ = [55°, 69°] and an acquisition time of 20 s. The diffraction peak (110) of ferrite and (111) of austenite were fitted and evaluated with a Gaussian function in StressTex Calculator software (version 2.0.5) [30]. Volume fractions and lattice spacing were quantified analogous to previous investigations at the STRESS-SPEC instrument [10,24]. The change in iron lattice plane distance ($a_{c}-a_{0}$) at austempering stage was correlated to carbon uptake by the relationship in Equation (1) [31]. The lattice constant $a_{0}$ represents the initial state after the quenching to austempering temperature.

(1) $$\omega_{c}^{\gamma }=\omega_{c,0}^{\gamma }+\frac{a_{c}-a_{0}}{0.033}$$

The maximum soluble amount of C in austenite is commonly described by the empirical equation of Voigt and Loper (Equation (2)) [32]. This value is used as the starting C content $w_{c,0}^{\gamma }$ for ausferritization stage, as the C back diffusion during quenching is negligible and no phase transformation takes place.

(2) $$w_{c,0}^{\gamma }=\frac{T_{\gamma }}{420}-0.17*w_{Si}-0.95$$

The equation describes the maximum amount of C $w_{c,0}^{\gamma }$ reached during austenitization at temperature $T_{\gamma }$ and is basis for the calculation of total C solved in austenite during austempering. The only alloying element considered is Si. The C content in austenite at 900 °C calculates to 0.79, 0.793 and 0.788 mass % for M1–M3, respectively. Out of several studies, Chang [33] derived a regression model for C uptake to improve accuracy and include the effect of alloying Mn, Ni, Cu and Mo. Using his model leads to values according to Table 2. However, we applied the model of Voigt to remain consistent with our previous work [10,24,34,35].

During the austempering at 300 and 350 °C, the maximum C content $\omega_{max}^{\gamma }$ was not reached within 2.5 h. The evolution of C content in austenite was of asymptotic shape. Therefore, an abortion criterion was used to compare the influence of Mo content for all investigated temperatures. As the second derivative of the C content evolution is smaller than $10^{-4}$, $\omega_{max}^{\gamma }$ is calculated.

### 2.5. In-Situ Dilatometry

The macroscopic length change during austenitization and austempering was measured with a DIL 805A/D quenching dilatometer from TA Instruments. Some minor modifications as an entrance and exit window for incident neutron beam and scattered neutrons enabled the simultaneous acquisition of neutron diffraction and dilatometric data. The “Alpha” measuring unit was utilized to achieve a length change resolution of 10 nm. The dilatometer samples are of cylindrical shape with 5-mm diameter and 10 mm in length.

### 2.6. Atom Probe Tomography

The sample for APT measurements on M2 material with 0.25 mass % Mo was prepared after heat treatment at 400 °C for 2030 s by means of polishing up to 1 μm and etching with 3% Nital. The material for the tips were extracted utilizing an Auriga 60 FIB (Carl Zeiss AG, Oberkochen, Germany) with attached OmniProbe manipulator (Oxford Instruments, High Wycombe, UK) and positioned at the prepared coupon for further thinning and shaping by focused ion beam. After the FIB preparation, the needles were channeled in the LEAP 4000X HR APT (CAMECA SAS, Gennevilliers, France) and analyzed at 70 K and an atmospheric pressure of $10^{-9}$ bar under high voltage field in laser mode with 30–50 pJ pulse energy. The atom positions were reconstructed and analyzed with CAMECA IVAS 3.8.2 Software using either SEM images when available or adjusting plane distances to match expectation by implying a spatial distribution map algorithm called AtomVicinity enhanced with auto-correlation [36].

**Figure 2:**
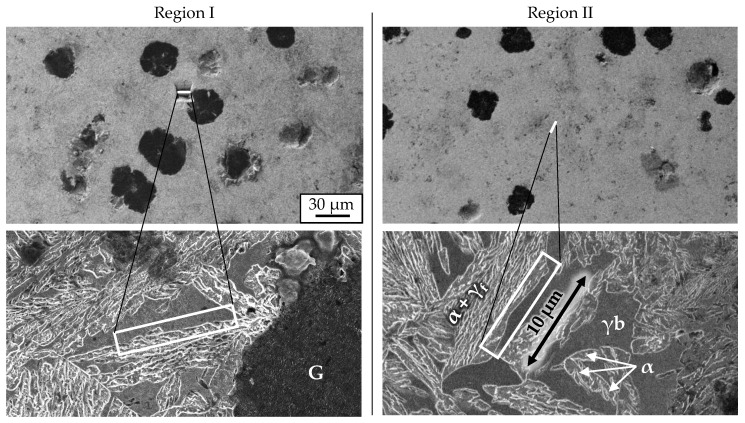
The two extraction regions are shown. The white rectangle area marks the boundaries of extracted material. Graphite (G), blocky austenite ($\gamma_{b}$), ferrite (α) and film-like austenite ($\gamma_{f}$) in between the ferrite form the microstructure.

Two extraction regions were chosen to see the effects of Mo segregation. Region I is located close to graphite nodule while Region II lies in a former pearlitic domain in the intercellular region. The main criteria for local region alignment was to comprise the interface between blocky austenite and ferrite. The finally carved out needle length axis orientation is perpendicular to the paper plane in Figure 2.

For atom concentration quantification, proximity histograms—short “proxigrams”—of the marked investigation iso-concentration surfaces were used (Figure 11), as described by Hellman et al. [37,38]. A proxigram shows the average concentration of each element orthogonal to the iso-concentration surface. The method was implemented to investigate segregation within interfaces independent of the interface geometry.

## 3. Results and Discussion

The results from neutron diffraction, metallography, dilatometry and atom probe tomography are presented and discussed along the ADI heat treatment route from initial material state, austenitization, to austempering. The first task during ADI heat treatment is achieving a complete transformation of cementite and ferrite into an austenitic structure and its full saturation with carbon. The maximum carbon content in austenite and the necessary enrichment time depend on the chosen austenitization temperature and the initial spatial distribution of carbon atoms in the microstructure. Present alloying elements that are solved in the iron matrix accelerating or impeding C-diffusion also have to be taken into account. The allocation of C within the initial material is represented by the size, shape and count of graphite nodules as well as the amount and distribution of pearlite (cementite). These carbon sources define the diffusion distances. The amount of carbon solved in initial ferrite is below 0.02 mass %. Darwish [39] found times between 2 and 60 min coming either of a pure pearlitic or ferritic microstructure until carbon enrichment is completed. Lee et al. [40] showed in their review that Mo retards carbon diffusion within the iron lattice.

### 3.1. In-Situ Neutron Diffraction

Our neutron diffraction data confirm that the phase transformation of initial phases to austenite at 900 °C is fast compared to the carbon enrichment. After 30 min, it is completed for M1–M3 samples.

A fully C saturated austenitic microstructure marks the end of austenitization and the quenching step follows to achieve an ausferritic microstructure. The sample consists of graphite nodules (about 11.5 vol.%) and austenitic matrix (about 88.5 vol.%). Only the evolution of austenite phase is followed with neutron diffraction and the austenite matrix is therefore treated initially as 100% (=1).

Stage 1 comprises the transformation from austenite into acicular ferrite and retained austenite (ausferrite) until the reaction slows down significantly and reaches a plateau. The plateau start time $t_{pl,start}$ represents the beginning of the process window. At this time, the formation of ferrite needles is completed and the diffusion of carbon is still in progress. The austenite plateau start time $t_{pl,start}$ and plateau austenite volume fraction $\Phi_{pl}^{\gamma }$ were determined in respect of a lower bound in transformation rate of $-10^{-1}$ according to Saal [10].

**Figure 3:**
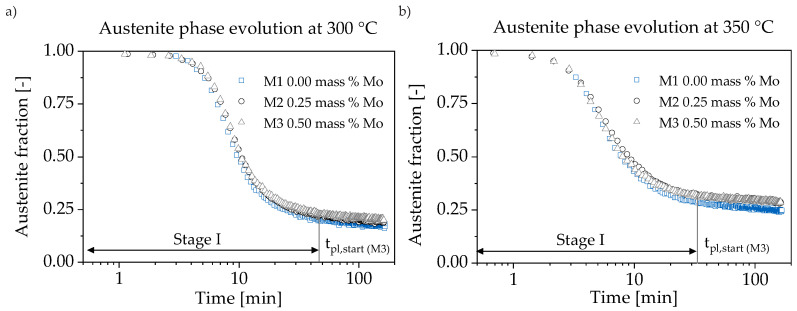
The phase transformation during austempering is depicted. (**a**) At 300 °C and (**b**) 350 °C, the decomposition of retained austenite is too slow to derive plateau end times within 2.5 h. Therefore, no impeding effect on carbide formation of Mo is indicated.

Austempering at 300 and 350 °C (Figure 3) is accompanied with an almost stable behavior of retained austenite after $t_{pl,start}$. The beginning Stage II reaction consumes about 3% of austenite within 2 h at both 300 and 350 °C. The Mo content variation has almost no influence on Stage I reaction speed. Adding 0.25 mass % Mo increases at 300 °C the retained austenite level by about 1.5% in M2 and M3. This stands in contrast to the effect occurring at 350 °C where the retained austenite levels of M2 and M3 coincide. They are 3% above M1 from the beginning. In comparison to the 300 °C step, the elevated temperature (350 °C) in Figure 3b contracts the Stage I reaction from 45 to 33 min. In addition, it shows a 10% higher retained austenite fraction.

**Figure 4:**
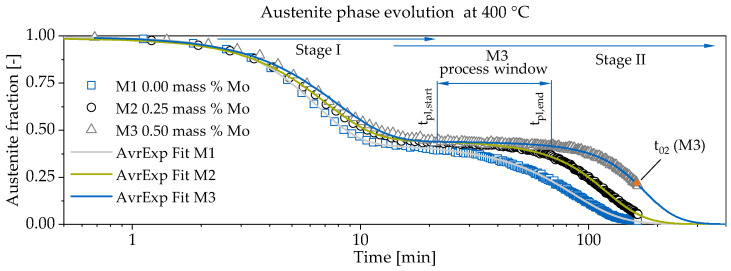
The phase transformation during austempering is depicted. At 400 °C, the influence of Mo content variation is resulting in the elongation of the process window. The $t_{pl,start}$ and $t_{pl,end}$ (process window) is exemplary depicted for M3 material.

**Figure 5:**
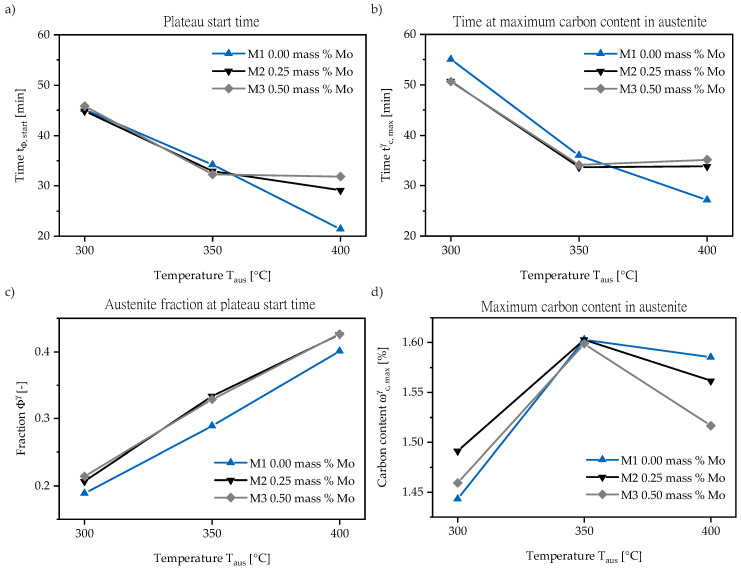
(**a**) The necessary time $t_{pl,start}$ for complete microstructure transformation is depicted and (**c**) the corresponding retained austenite fraction is presented. The dependency of maximum carbon content of austempering temperature can be followed (**d**), while the therefore necessary time is presented (**b**).

Figure 4 depicts the evolution of austenite to ausferrite phase transformation during austempering at 400 °C for M1–M3. For all materials (M1–M3), a continuous decomposition of retained austenite can be followed after reaching $t_{pl,start}$. The addition of 0.25 and 0.50 mass % Mo slows down the Stage II reaction and opens the heat treatment process window, which is exemplarily shown for M3. As summarized in Table 3, the austenite to ausferrite transformation is retarded and the plateau point $t_{pl,start}$ is reached later. The amount of retained austenite increases by about 3% (Figure 5).

The acquired data represent two different transformation mechanisms taking place during Stages I and II. Therefore, a combination of two e-functions is used to fit the evolution of austenite volume fraction $\Phi_{\gamma }$ according to Equation (3). Further, this combination enables to model a start of Stage II reaction before Stage I reaction is completed.

(3) $$\Phi_{\gamma }\left(t\right)=y_{0}+A\;\cdot \;\left(p\;\cdot \;e^{-k_{1}\;\cdot \;t^{n}}+\left(1-p\right)\;\cdot \;\frac{1}{1+e^{k_{2}\left(t-t_{02}\right)}}\right)$$

The first exponential function is an Avrami equation with its exponent *n* and factor $k_{1}$ [41]. In its original form, it describes the growth of a new phase. As we follow the decreasing austenite fraction, the original equation is adjusted accordingly. The assumptions for a classic phase transition after Johnson, Mehl, Avrami and Kolmogorov (JMAK), such as homogeneous nucleation, are not granted to derive physical parameters like a nucleation rate. However, the exponent *n* gives some information on the reaction modality. Following the conditions reported by Christian [42], a decreasing value of n around 2 indicates full saturation of point nucleation sites and a transition from grain edge nucleation to grain boundary nucleation. With Equation (4), the relaxation time τ can be derived from $k_{1}$. It is independent of *n* and represents the time at which 63.2% of Stage I reaction is completed.

(4) $$\tau =k_{1}^{-\frac{1}{n}}$$

The results are listed in Table 4. With increasing Mo amount, the Avrami exponent *n* is lowered from 2.3 to 1.9. The slowed down reaction kinetics is also reflected in an increase of relaxation time from 6.38 to 7.81 min. Accordingly, the maximum transformation speed is found in M1. The descent of *n* indicates a more heterogeneous nucleation. The proportion *p* links the JMAK expression with a more phenomenological approach. A sigmoidal logistic function is chosen to fit the austenite fraction during Stage II. The amplitude *A* is set to 1 and the basis $y_{0}$ equals 0 to describe a complete decay of austenite. The fit allows to derive the maximum decay speed ${\dot{\Phi }}_{\gamma ,max}$ in the infliction points at $t_{02}$. Further, the data is extrapolated for complete decay time $t_{cD}$ estimation (i.e., $\Phi_{\gamma }$ has reached 2.5%). The results of Stage II at 400 °C are summarized in Table 5.

Concerning the amount of retained austenite reached, it can be stated that Mo leads to an 2–3% increase in retained austenite fraction at all temperatures. Figure 5c reveals that for M2 the maximum retained austenite fraction gain is already reached and the addition of further 0.25 mass % Mo (M3) has no significant effect. This stands in contrast to the prolongation of the process window, where the duration seems to be directly proportional to the added Mo amount within the investigated range. More investigations with different Mo contents are necessary to characterize the nature of the relationship between process window duration and Mo content, because the conduction of this relationship is strongly dependent on chosen abortion criteria. The achieved amount of retained austenite after Stage I is a function of austempering temperature, initial and final C content in retained austenite and the influence of the alloying element Mo itself. Lower initial C content is a result of Mo addition if Chang’s model is considered. Less C in austenite is linked to a decrease in its stability and would have been accompanied with a decrease in the retained austenite volume fraction at higher Mo contents. Since the opposite is the case, it can be assumed that the stabilizing effect caused by the Mo itself slightly overcompensates the lower C content.

Figure 5 summarizes the neutron diffraction results for all austempering temperatures and reveals a maximum in austenite carbon content at 350 °C.

**Figure 6:**
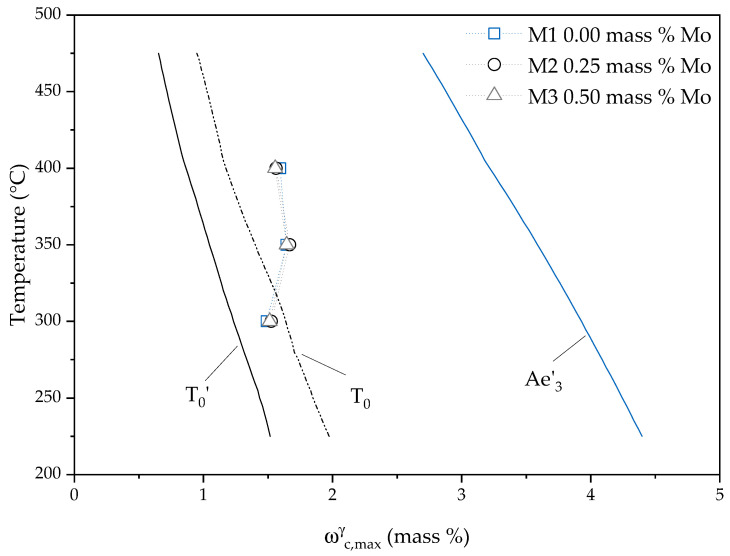
The maximum C contents of austenite found during austempering in M1–M3 material are compared to the thermodynamically calculated values for the equilibrium state $A_{e3}^{\prime }$ and to the theoretical values achieved with the displacive mechanism—$T_{0}$ and $T_{0}^{\prime }$. $T_{0}^{\prime }$ takes an additional 400 J/mol distortion energy stored in ferrite into account.

As the redistribution of C from ferrite to austenite is a diffusive process, the maximum amount of C in austenite is reached a short time after the final formation of microstructure, as shown in Figure 5a,b. This supports the theory of a displacive mechanism for the creation of lower bainitic ferrite, as proposed by Bhadeshia [43]. If the process had been primarily diffusion controlled, the end of ausferrite formation and time of maximum C content would have coincided. To investigate this in more detail, an analysis was performed to calculate $T_{0}$, $T_{0}^{\prime }$ and $A_{e3}^{\prime }$ concentrations [44]. Figure 6 shows the result combined with the determined maximum C content of austenite within the investigated alloys. The influence of Mo alloying on the maximum C content is negligible compared to the temperature effect as described above. The temperature mainly influences how the final ADI microstructure (i.e., fineness of acicular ferrite, retained austenite content and shape) is formed. Regarding austempering at 300 °C the results are in good agreement with Bhadesia’s theory, especially when taking $T_{0}^{\prime \prime }$ into account, which is located between $T_{0}^{\prime }$ and $T_{0}$ [45]. For elevated austempering temperatures the results are slightly shifted towards the equilibrium $A_{e3}^{\prime }$ line but are still close to the $T_{0}$ curve. The current neutron diffraction measurements average over a volume of 5 × 5 × 5 mm³. Here, one might reason that for elevated temperatures locally both effects take place. The mobility of C is much higher at that temperatures and C might be more quickly distributed over the larger blocky austenite areas. However, the maximum C enrichment values for $T_{aus}$ = 400 °C and $T_{aus}$ = 350 °C are near the $T_{0}$ line, which indicates that displacive transformation seems to be dominant.

### 3.2. Metallography

**Figure 7:**
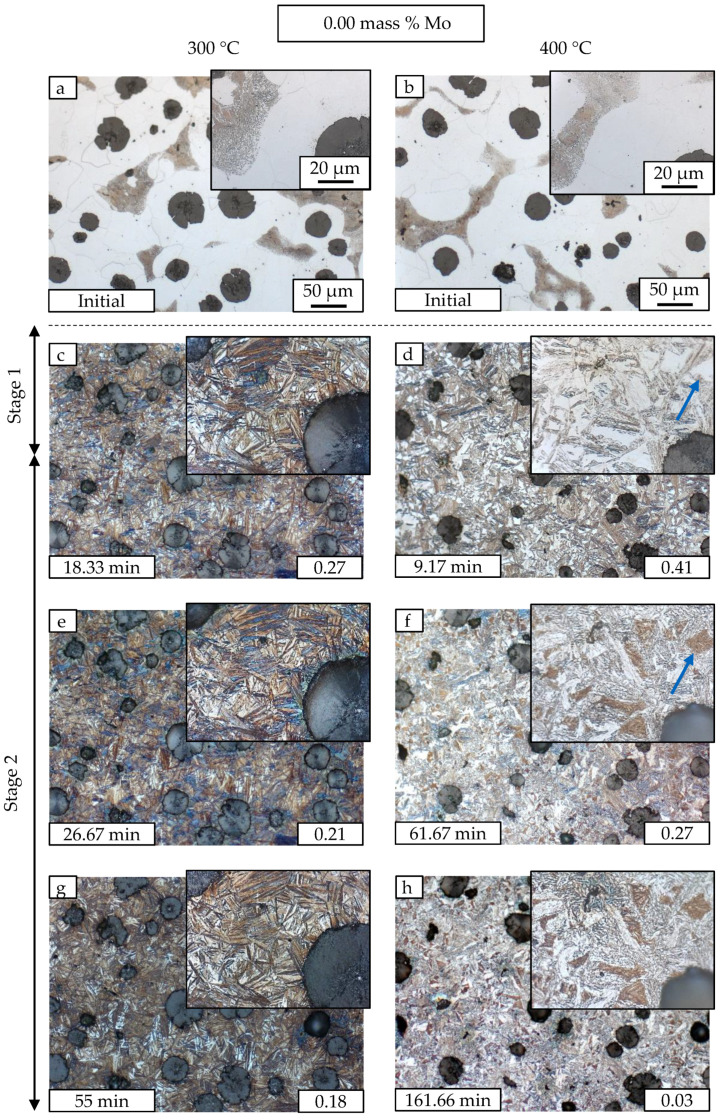
The evolution of austenite decomposition over time for M1 is shown by Nital etched LOM images. The austempering temperatures are 300 °C (**c**,**e**,**g**) and 400 °C (**d**,**f**,**h**). The initial material state is depicted in (**a**,**b**). The time labels represent the extraction time and are chosen to match the retained austenite content. The austenite fraction is depicted at the lower right corner of each micrograph.

**Figure 8:**
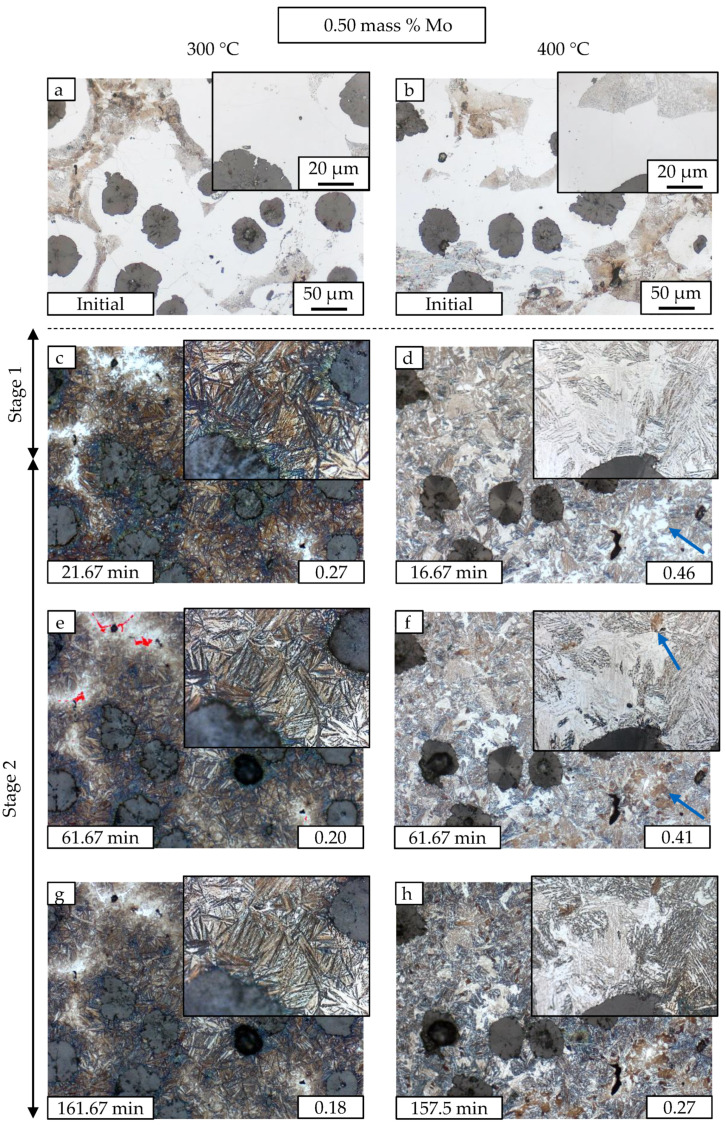
The evolution of austenite decomposition over time for M3 is shown by Nital etched LOM images. The austempering temperatures are 300 °C (**c**,**e**,**g**) and 400 °C (**d**,**f**,**h**). The initial material state is depicted in (**a**,**b**). Carbides in (**e**) are colored in red.

In addition to neutron diffraction results, the ausferrite microstructure evolution is investigated with metallography. The heat treatment method introduced in Section 2.3 is utilized to follow the microstructure at 300 and 400 °C at the same sample location. Figure 7 and Figure 8 show micrographs at several austempering times for M1 and M3, respectively. The quasi stable behavior at 300 °C for 2 h and the decomposition process at 400 °C can be followed. Figure 7 is divided into two columns. The conditions for 300 °C are shown on the left, while for 400 °C on the right. For both temperatures, the initial states before ADI heat treatment are depicted first, which have the classic structure of ductile iron. The graphite nodules are surrounded by ferrite and in the intermediate areas pearlite has formed after solidification. A detailed section illustrates the changes in microstructure in each case. The ADI heat treatment process is carried out until the correspondingly marked austempering time is attained. Where possible, the chosen extraction times compare states with same amount of retained austenite. The austenite fraction is depicted at the lower right corner of each micrograph. The first micrographs with ADI microstructure (Figure 7c,d) show the well-known difference in ferrite needle morphology. Very fine brown ferrite needles in large numbers at 300 °C with small white retained austenite areas in between are opposed to retained austenite areas with several μm in diameter (blocky austenite) and larger ferrite plates at 400 °C. Higher supercooling at 300 °C leads to more ferrite nucleation points and results in finer microstructure. After reaching the plateau end time $t_{pl,end}$, the retained austenite starts to decompose into carbides and ferrite. There are blocky austenite regions which shift from white to brown color (marked with arrows in Figure 7d,f). We propose that this shift indicates the formation of first carbides during Stage II reaction. Note that the two states have a difference of 14% in retained austenite content. The austenite stability is reduced during decomposition as it gets depleted of carbon caused by the formation of carbides. An other explanation would be martensite formation. The first image taken (Figure 7d and Figure 8d), however, was in all cases free of brownish blocky austenite, proving a high enough enrichment with carbon and therefore sufficient stability against thermal induced martensite formation. The effect is also present in M3 material but less distinctive. The micrographs of M3 in Figure 8 show mixture carbides rich in Mo and Mn that are forming in the intercellular regions stemming from Mo segregation during solidification. The carbides are not dissolved during the austenitization at 900 °C. For better visualization, the mixture carbides are colored in red in (Figure 8e).

Neutron diffraction clarifies that most of the white areas in M1 at 400 °C and 162 min (Figure 7h) are no longer retained austenite but almost entirely ferrite. The evaluation of austenite and ferrite peak intensities is resulting in less than 2% of austenite at this time. The micrographs support the in-situ neutron diffraction results and give insight in the microstructure development. The micrographs give a clear impression of the spatial arrangements of different precipitation products during annealing. However, for the quantitative evaluation of the respective fractions, neutron diffraction is the method of choice as reliable ensemble averages over the whole bulk sample are given.

### 3.3. Comparison of Neutron Diffraction to Dilatometry

**Figure 9:**
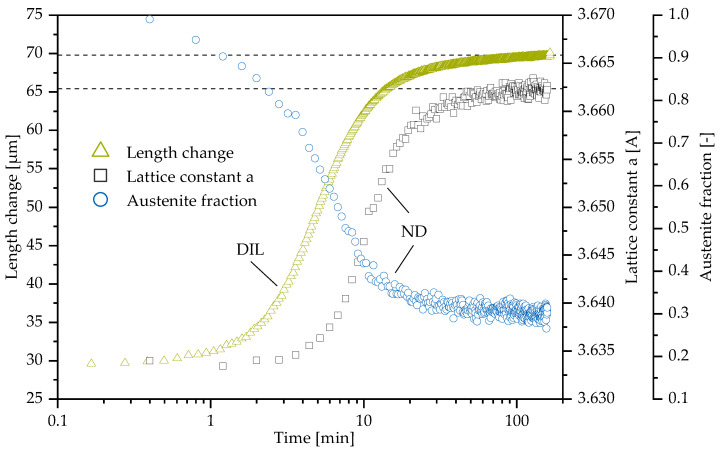
The austempering temperature is 350 °C. The evolution of retained austenite decomposition over time for M3 is analyzed with neutron diffraction and dilatometry. The macroscopic length change can be followed as well as the peak position shift due to C uptake. Neutron diffraction (ND) and dilatometry data (DIL) are marked, respectively.

Dilatometry is a well-established method for phase transition investigations. The combined operation on the STRESS-SPEC instrument allows both the recording of diffractograms and macroscopic length change during the heat treatment simultaneously. Figure 9 shows the comparison of macroscopic length change to the results from neutron diffraction at an ausferritization temperature of 350 °C for M3 material. The evolution of austenite phase volume fraction is discussed in the previous section. In addition, the course of austenite lattice constant is shown—its increase is associated with the uptake of C in austenite. The lattice constant change is derived out of the peak position shift of the austenite (111) peak. While macroscopic length and austenite phase volume fraction show increasing rates after 1 min, the increase of austenite lattice constant starts slightly delayed. This is as the diffusion process of C atoms, which move into austenite after the formation of ferrite, requires time.

Note that, in our earlier work ([24] and more recently also [46]), we showed that different enrichment of austenite with C during the ausferrite transformation process is visible in asymmetries of the (111) reflection. This peak asymmetry could be described with two separate peaks. However, Meier et al. [24] showed that the peak analysis with a single Gaussian function yields essentially the same transformation trends as a multi-peak analysis. In the current case, we found slight peak shape asymmetries only between transformation times of around 3 and 8 min. Before and after, no significant deviation from a single Gaussian peak occurred. This indicates that the trends can be correctly approximated using a single Gaussian function, which we therefore kept for comparability to former results [10,47].

In the first few minutes, the macroscopic length change is therefore primarily influenced by the transformation from austenite to ferrite. Subsequently, the change in length results both from phase transformation and C uptake in the remaining austenite. The values recorded with dilatometry and neutron diffraction should converge to a maximum, which is not reached within the period under investigation. In contrast to 400 °C, the maximum stabilization of retained austenite is not yet achieved because a further enrichment with C still takes place.

**Figure 10:**
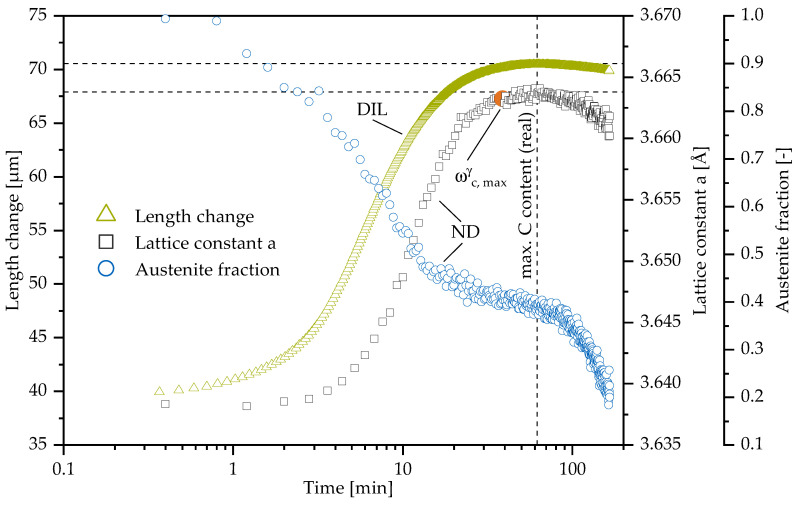
The austempering temperature is 400 °C. The measured length change data from dilatometry compared to the neutron diffraction results. Maximum carbon enrichment and maximum length change are marked as well as the data origin—neutron diffraction (ND) and dilatometry (DIL).

At 400 °C (Figure 10) the accelerated reaction kinetic allows us to follow the decomposition of highly C enriched austenite to carbides and ferrite. Two maximum C saturation times are present in the figure. The first is derived from curve fitting and abortion criteria described earlier to compare the value with lower austempering temperatures. The second represents the real maximum C content time at 400 °C. The real maximum in C content matches the maximum length change and marks the beginning Stage II reaction. The influence of ferrite formation on macroscopic sample dimensions divides into two domains, first from beginning to end of ausferrite formation ($t_{pl,start}$) and second from there until no austenite is left.

The starting decomposition of retained austenite should have resulted in a positive length change, as it did before during the transformation to ausferrite, but the length change decreases and seems to follow the development of austenite C content instead. One explanation would be that the effects contributing to the macroscopic length change (namely, possible change in C content of austenite, ferrite and carbide formation) are leveling each other out. The change in austenite fraction after reaching the maximum C content should have resulted in about 5 μm positive length change due to formation of ferrite. As no positive macroscopic length change is observed, this would have to be achieved through the decrease of the austenite lattice constant alone (note that ferrite and carbide formation result in a positive length change, as seen for instance during pearlite formation [48]). However, the observed change in lattice constant is too small to compensate the expected macroscopic length change.

Therefore, as a possible alternative mechanism, we propose that the acicular ferrite is forming a stable network at the end of Stage I and further decomposition of encapsulated retained austenite regions only causes local stresses but does not have much effect on the sample dimensions anymore. In contrast to Stage I the further creation of ferrite during Stage II does not enrich the present austenite additionally with C. Rather, the excess C from austenite to ferrite phase transition as well as C from the remaining high carbon austenite is consumed to form iron carbides or diffuses back to the nodules and the observed decrease in austenite lattice constant therefore is mainly due to local stresses.

In contrast to neutron diffraction results, the end of transformation from full austenitic to ausferritic microstructure cannot be derived from dilatometry data alone. Thus, neutron diffraction gives a more detailed insight by enabling the measurement of phase quantity evolution (from the measured intensity) apart from enrichment of carbon (from the measured 2-Θ peak shift). However, dilatometry allows finding the time of maximum carbon enrichment of austenite for austenitization and ausferritization and makes it a valuable method to identify the end of the process window.

### 3.4. Atom Probe Tomography (APT)

**Figure 11:**
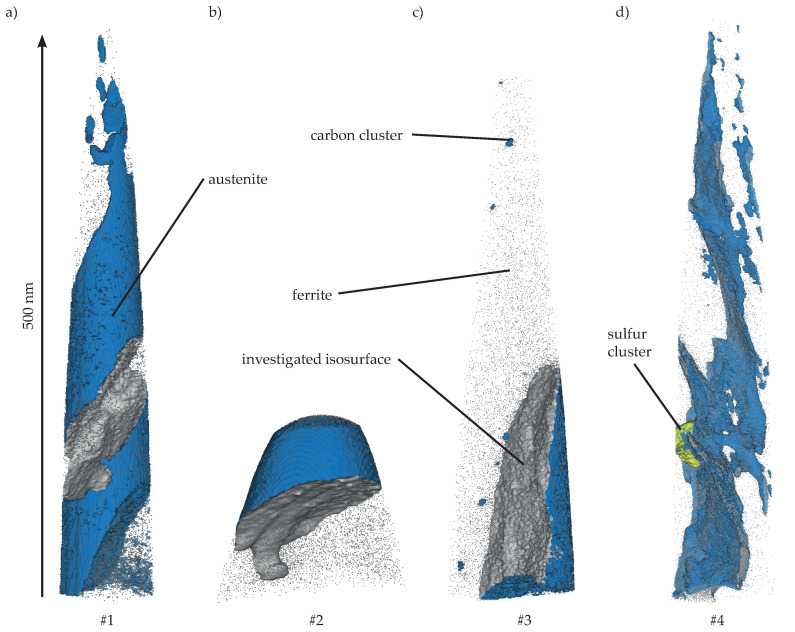
(**a**–**d**) The investigated tips are presented. The austenite is enclosed by a blue iso-concentration surface and the basis of the proxigram interface analysis is represented by the gray iso-concentration surface. Tips #1 and #2 are extracted close to a graphite nodule (Region I). Tips # 3 and # 4 are taken from a position of former pearlitic microstructure (Region II). A small sulfur cluster is contained in Tip # 4 and marked with a yellow iso-concentration surface.

Next, we investigated M2 material at maximum carbon content with APT focusing on the interface of ferrite and retained austenite as well as the distribution of C, Si, Mn and Cr. An overview of the measured tips is given in Figure 11. Tip extraction points are presented in Figure 2. Tips # 1 and # 2 are extracted from Region I close to a nodule. Information from Region II is represented by Tips # 3 and # 4. A blue iso-concentration surface with a boundary value of 5.3 at.-% C divides austenitic from ferritic domains visually. In Tip # 1 the austenite is pierced by several ferrite lathes while Tip # 2 has a single austenite/ferrite boundary layer. In addition to the transition from face-centered to body-centered cubic lattice, individual carbon clusters in the ferrite were identified in the third tip. Austenite areas in Tips #1–3 were mostly closed, whereas in the last tip, Tip # 4, only thin austenite films exist between the ferrite.

**Figure 12:**
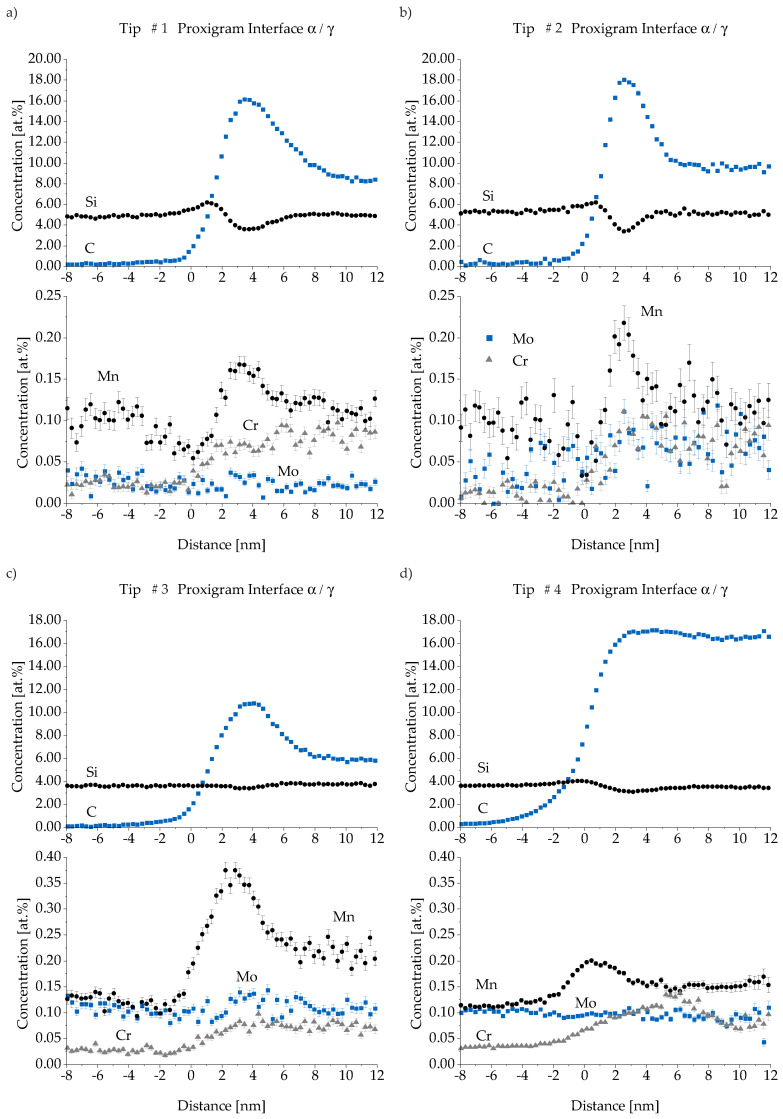
Proxigrams for Tips # 1–4 (from (**a**–**d**)) give insight in the enrichment of carbon in the interface between austenite and ferrite. For better scaling, concentrations of C and Si are plotted separately from Mo, Mn and Cr. Tip # 1 in (**a**) and Tip 2 in (**b**) are from Region I. Region II is represented by Tip # 3 in (**c**) and Tip # 4 in (**d**).

A local profile analysis of each carbon clusters in Tip # 3 reveals a maximum concentration of 12 at.-% C for every cluster. Their existence indicates an ausferrite formation mechanism described for steel as lower bainite [49]. The diffusion rate of C is not sufficient to completely transport the interstitial element into austenite during the growth of ferrite tips. Therefore, the precipitation of iron carbide (25 at.-% C) in lower bainite occurs within the ferrite tips, which is probably not possible in the present ADI material due to the high silicon content. The cluster positions indicate iron lattice distortions, as C has the tendency to accumulate around them as experimentally proved most recently [50]. The proxigrams in Figure 12 show a carbon content of less than 0.3 at.-% for all ferrite regions. The austenite domains display various contents of C from 5.9 at.-% in Tip # 3 to 16.6 at.-% in Tip # 4 according to their macroscopic dimension.

An enrichment of C in the interface zones up to 18 at.-% followed by a lower C level in austenite can be reported for all tips except Tip # 4, where the amount of C stays constant after reaching its maximum. The latter represents the final state of film-like austenite that becomes finally iron carbide in case of accumulating enough carbon. Caballero [51] proved the precipitation of cementite within the austenite/ferrite interface in a nanostructured bainitic steel. As described, the atomic carbon concentration of 25% is not reached. This stands in contrast to earlier work, where the existence of cementite in ADI with comparable Si amount was documented [35].

One possible explanation would be the absence of cementite phase due to the high silicon amount in ductile iron. The solubility of Si in cementite is very low and as a consequence it has to diffuse away from cementite formation areas. This is further supported by Figure 12 showing the lowest Si concentration within the interface where C has its maximum. A more local ROI analysis finally confirmed the existence of cementite at least within the austenite film in Tip # 4, while the interface in Tip #3 can be seen as “cementite-free”. It also has to be mentioned that the carbon content measurement with APT, especially in the case of carbides, underestimates the real present value by up to 24%, because not all atoms can be detected properly. The carbon tends to be relieved in molecules and multiple events that follow in very short intervals. Current detectors may not be able to resolve the individual events properly and detector dead time is limiting the counts of carbon ions [52]. Therefore, a C concentration of 19% can be considered as cementite. Regarding the Si level from ferrite over the interface to austenite a slight increase of Si exists after entering the interface zone. It is followed by the Si minimum value matching the maximum C content. Austenite and ferrite have almost the same Si concentration. The mean Si level close to a nodule is 5.1 at.-% while Region II shows 3.8 at.-% —compare Figure 12a,b with Figure 12c,d. This fits to the bulk material value—4.4 at.-% Si (3.35 mass %)—and the well known positive segregation of Si. It is also in good agreement with electron micro probe analyzer (EPMA) measurements at ductile iron [20] where equivalent regions show Si levels of 5 at.-% and 3.8 at.-%, respectively.

There is no Cr enrichment in the interface but higher concentration levels are reached in austenite than in ferrite. Mo and Mn are both known for impeding the Stage I reaction but seem to use two different mechanisms. Mn, similar to C, shows higher concentrations in the alpha/gamma phase boundary, while the amount of dissolved Mo stays constant through the different phases. Mn may have an additional impact in slowing down the growth of ferrite needles due to its enrichment in the interface. The evaluated amount of Mo represents only 59% of the total Mo atoms because of a peak overlap in the mass-to-charge-spectra of molybdenum isotopes 96 and 98 with the 4C molecule. Therefore, both Mo isotopes were not taken into account, which add up to 41% of the natural isotope abundance of Mo according to Meija et al. [53]. The intensity of observed Mo isotopes peaks at higher charge states without overlapping is in excellent accordance with the natural abundance and make a partial evaluation valid. Comparing Region I to Region II, a four times higher Mo amount in the latter is occurring, which again is in good accordance with the segregation behavior of this element.

## 4. Conclusions

Mo slows down carbon diffusion and suppresses the formation of cementite. The Stage II reaction is not entirely shifted to longer times but slowed down enough to open the heat treatment process window.

The LOM investigations of interrupted heat treatment gave insight into the microstructure evolution and showed the coarsening of microstructure. However, our neutron diffraction data indicate that, in addition to metallography, high resolution SEM or EBSD are necessary to prevent misinterpretation of the “white” areas in the images. Dilatometry is a well-established method that is capable of determining the time for maximum C enrichment in ADI during austempering. That is very important for determining the finish time in the industrial heat treatment process. However, as neutron diffraction data reveal, dilatometry is not capable of defining the exact value for $t_{pl,start}$ and the decomposition of austenite in Stage II reaction cannot be followed adequately. The macroscopic length change during austempering is a combination of microstructure evolution and the carbon diffusion process. The latter has the bigger impact. APT data show that Mo is not enriched in the interface zone of austenite and ferrite. The measured Mo amount follows segregation theory and was found locally four times higher in Region 2 than in Region 1. The interface analysis and comparison of austenite and ferrite showed that Mo distribution is unaffected by phase change from austenite to ferrite. Carbon gets enriched in the interface until the growth of ferrite needles has progressed and the resulting austenite lath in between matches the carbon content of the interface and finally becomes cementite. The proof of carbide formation out of former retained austenite regions is planned in further APT measurements.

## Figures and Tables

**Table 1:** Material compositions in mass %.

Material	C	Si	Mn	Mo	Ni	Cu	Mg	S	P	Ti	Fe
M1	3.73	2.37	0.18	0.00	0.02	0.02	0.05	0.01	0.04	0.01	bal.
M2	3.68	2.35	0.18	0.25	0.02	0.03	0.04	0.02	0.04	0.01	bal.
M3	3.63	2.38	0.18	0.49	0.02	0.03	0.04	0.01	0.04	0.01	bal.

**Table 2:** Initial carbon content $w_{c,0}^{\gamma }$ in austenite.

Material	Voigt [32]	Chang [33]
	[mass %]	[mass %]
M1	0.790	0.704
M2	0.793	0.675
M3	0.788	0.645

**Table 3:** Characteristic process window values and time of maximum C content for austempering at 400 °C.

Material	*t_pl,start_*	*t_pl,end_*	Duration	$t_{c,\max}^{\gamma }$
	[min]	[min]	[min]	[min]
M1	17.09	27.36	10.27	27.15
M2	21.57	42.89	21.32	33.85
M3	27.22	72.51	45.29	35.15

**Table 4:** Fitting parameters and derived values for Stage I.

Material	Proportion *p*	Relax. Factor $k_{1}$	Relax. Time τ	Max. Transf. Speed ${\dot{\Phi }}_{\gamma ,\max}$	Avrami Exp. *n*
	[-]	[-]	[min]	[%/min]	[-]
M1	0.5371	0.0137	6.38	−8.45	2.315
M2	0.5350	0.0191	7.03	−6.64	2.029
M3	0.5529	0.0208	7.81	−5.86	1.884

**Table 5:** Fitting parameters and derived values for Stage II.

Material	Relax. Factor $k_{2}$	Infliction Point $t_{02}$	Max. Decay Speed ${\dot{\Phi }}_{D,\max}$	Complete Decay $t_{\mathrm{cD}}$
	[-]	[min]	[%/min]	[min]
M1	0.0353	66.45	−0.424	148.64
M2	0.0340	104.32	−0.395	188.79
M3	0.0279	160.05	−0.312	261.21

## References

[1] Boccardo A.D., Dardati P.M., Godoy L.A., Celentano D.J. (2018). Sensitivity of Austempering Heat Treatment of Ductile Irons to Changes in Process Parameters. Metall. Mater. Trans. B Process Metall. Mater. Process. Sci..

[2] Gazda A., Warmuzek M. (2018). Analysis of thermal stability of ausferrite obtained by means of cyclic heat treatment of Cu–Ni (Mn, Mo) ADI. Thermochim. Acta.

[3] Eric Cekic O., Rajnovic D., Sidjanin L., Janjatovic P., Balos S., Mitrovic N., Mitrovic M., Mitrovic G. (2020). Dual Phase Austempered Ductile Iron—The Material Revolution and Its Engineering Applications. Computational and Experimental Approaches in Materials Science and Engineering.

[4] Górny M., Angella G., Tyrała E., Kawalec M., Paź S., Kmita A. (2019). Role of Austenitization Temperature on Structure Homogeneity and Transformation Kinetics in Austempered Ductile Iron. Met. Mater. Int..

[5] DIN Deutsches Institut für Normung e. V. (2012). Gießereiwesen—Ausferritisches Gusseisen mit Kugelgraphit.

[6] Laino S., Sikora J.A., Dommarco R.C. (2008). Development of wear resistant carbidic austempered ductile iron (CADI). Wear.

[7] Masaggia S. (2010). The development of ADI and IDI in Italy. Fatigue.

[8] Daber S., Ravishankar K.S., Prasad Rao P. (2008). Influence of austenitising temperature on the formation of strain induced martensite in austempered ductile iron. J. Mater. Sci..

[9] Daber S., Prasad Rao P. (2008). Formation of strain-induced martensite in austempered ductile iron. J. Mater. Sci..

[10] Saal P., Meier L., Li X., Hofmann M., Hoelzel M., Wagner J.N., Volk W. (2016). In Situ Study of the Influence of Nickel on the Phase Transformation Kinetics in Austempered Ductile Iron. Metall. Mater. Trans. A.

[11] Górny M., Tyrała E., Sikora G. (2018). Transformation Kinetics and Mechanical Properties of Copper-Alloyed and Copper-Nickel Alloyed ADI. Mater. Sci. Forum.

[12] Benam A.S. (2015). Effect of alloying elements on austempered ductile iron (ADI) properties and its process: Review. China Foundry.

[13] Yazdani S., Elliott R. (1999). Influence of molybdenum on austempering behaviour of ductile iron Part 4—Austempering behaviour of ductile iron containing 0·45%Mo. Mater. Sci. Technol..

[14] Yazdani S., Elliott R. (1999). Influence of molybdenum on austempering behaviour of ductile iron Part 3—Austempering kinetics, mechanical properties, and hardenability of ductile iron containing 0·25%Mo. Mater. Sci. Technol..

[15] Yazdani S., Elliott R. (1999). Influence of molybdenum on austempering behaviour of ductile iron Part 2—Influence of austenitising temperature on austempering kinetics, mechanical properties, and hardenability of ductile iron containing 0·13%Mo. Mater. Sci. Technol..

[16] Yazdani S., Elliott R. (1999). Influence of molybdenum on austempering behaviour of ductile iron Part 1—Austempering kinetics and mechanical properties of ductile iron containing 0·13%Mo. Mater. Sci. Technol..

[17] Dekker L., Tonn B. (2016). Occurrence and behaviour of Mo containing precipitates in nodular cast iron at high temperatures. Int. J. Cast Met. Res..

[18] Gazda A., Warmuzek M., Bitka A. (2018). Optimization of mechanical properties of complex, two-stage heat treatment of Cu–Ni (Mn, Mo) austempered ductile iron. J. Therm. Anal. Calorim..

[19] Dorazil E. (1991). High Strength Austempered Ductile Cast Iron.

[20] Domeij B. (2017). On the Solidification of Compacted and Spheroidal Graphite Irons.

[21] Caballero F.G., Miller M.K., Garcia-Mateo C., Cornide J., Santofimia M.J. (2012). Temperature dependence of carbon supersaturation of ferrite in bainitic steels. Scr. Mater..

[22] DIN Deutsches Institut für Normung e. V. (2016). Gießereiwesen—Gusseisen mit Kugelgraphit.

[23] DIN Deutsches Institut für Normung e. V. (2010). Mikrostruktur von Gusseisen—Teil 1: Graphitklassifizierung Durch Visuelle Auswertung (ISO 945-1:2008 + Cor. 1:2010): Deutsche Fassung EN ISO 945-1:2008 + AC:2010.

[24] Meier L., Hofmann M., Saal P., Volk W., Hoffmann H. (2013). In-situ measurement of phase transformation kinetics in austempered ductile iron. Mater. Charact..

[25] Hofmann M., Schneider R., Seidl G.A., Rebelo-Kornmeier J., Wimpory R.C., Garbe U., Brokmeier H.G. (2006). The new materials science diffractometer STRESS-SPEC at FRM-II. Phys. B Condens. Matter.

[26] Zeitelhack K., Schanzer C., Kastenmüller A., Röhrmoser A., Daniel C., Franke J., Gutsmiedl E., Kudryashov V., Maier D., Päthe D. (2006). Measurement of neutron flux and beam divergence at the cold neutron guide system of the new Munich research reactor FRM-II. Nucl. Instrum. Methods Phys. Res. Sect. A Accel. Spectrom. Detect. Assoc. Equip..

[27] Hofmann M., Gan W., Rebelo-Kornmeier J. (2015). STRESS-SPEC: Materials science diffractometer. J. Large-Scale Res. Facil. JLSRF.

[28] Rebelo Kornmeier J., Gibmeier J., Hofmann M. (2011). Minimization of spurious strains by using a Si bent-perfect-crystal monochromator: Neutron surface strain scanning of a shot-peened sample. Meas. Sci. Technol..

[29] Rebelo-Kornmeier J., Hofmann M., Gan W.M., Randau C., Braun K., Zeitelhack K., Defendi I., Krueger J., Faulhaber E., Brokmeier H.G. (2017). New Developments of the Materials Science Diffractometer STRESS-SPEC. Mater. Sci. Forum.

[30] Randau C., Garbe U., Brokmeier H.G. (2011). StressTextureCalculator: A software tool to extract texture, strain and microstructure information from area-detector measurements. J. Appl. Crystallogr..

[31] Roberts C.S. (1953). Effect of Carbon on the Volume Fractions and Lattice Parameters of Retained Austenite and Martensite. JOM.

[32] Voigt R.C., Loper C.R. (1984). Austempered Ductile Iron—Process Control and Quality Assurance. J. Heat Treat..

[33] Chang L.C. (2003). An analysis of retained austenite in austempered ductile iron. Metall. Trans. A.

[34] Li X.H., Saal P., Gan W.M., Hoelzel M., Volk W., Petry W., Hofmann M. (2017). Strain-Induced Martensitic Transformation Kinetic in Austempered Ductile Iron (ADI). Metall. Mater. Trans. A.

[35] Li X., Wagner J.N., Stark A., Koos R., Landesberger M., Hofmann M., Fan G., Gan W., Petry W. (2019). Carbon Redistribution Process in Austempered Ductile Iron (ADI) During Heat Treatment—APT and Synchrotron Diffraction Study. Metals.

[36] Boll T., Zhu Z.Y., Al-Kassab T., Schwingenschlögl U. (2012). Atom probe tomography simulations and density functional theory calculations of bonding energies in Cu_3_Au. Microsc. Microanal..

[37] Hellman O.C., Vandenbroucke J.A., Rüsing J., Isheim D., Seidman D.N. (2000). Analysis of Three-dimensional Atom-probe Data by the Proximity Histogram. Microsc. Microanal..

[38] Hellman O.C., Du Rivage J.B., Seidman D.N. (2003). Efficient sampling for three-dimensional atom probe microscopy data. Ultramicroscopy.

[39] Darwish N., Elliott R. (1993). Austempering of low manganese ductile irons: Part 2 Influence of austenitising temperature. Mater. Sci. Technol..

[40] Lee S.J., Matlock D.K., Van Tyne C.J. (2011). An Empirical Model for Carbon Diffusion in Austenite Incorporating Alloying Element Effects. ISIJ Int..

[41] Avrami M. (1941). Granulation, Phase Change, and Microstructure Kinetics of Phase Change. III. J. Chem. Phys..

[42] Christian J.W. (2002). The Theory of Transformations in Metals and Alloys.

[43] Bhadeshia H.K.D.H. (2015). Bainite in Steels: Theory and Practice.

[44] Lee J.L., Bhadeshia H. (1993). A methodology for the prediction of time-temperature-transformation diagrams. Mater. Sci. Eng. A.

[45] Hall D.J., Bhadeshia H.K., Stobbs W.M. (1982). The incomplete bainite reaction: Possible reasons for the apparent differences in TEM and atom probe determination of austenite carbon content. J. Phys..

[46] Onuki Y., Hirano T., Hoshikawa A., Sato S., Tomida T. (2019). In Situ Observation of Bainite Transformation and Simultaneous Carbon Enrichment in Austenite in Low-Alloyed TRIP Steel Using Time-of-Flight Neutron Diffraction Techniques. Metall. Mater. Trans. A.

[47] Li X.H., Saal P., Gan W.M., Landesberger M., Hoelzel M., Hofmann M. (2016). Strain Induced Martensitic Transformation in Austempered Ductile Iron (ADI). J. Phys. Conf. Ser..

[48] García de Andrés C. (2002). Application of dilatometric analysis to the study of solid–solid phase transformations in steels. Mater. Charact..

[49] Bhadeshia H., Edmonds D.V. (1980). The mechanism of bainite formation in steels. Acta Metall..

[50] Rementeria R., Domínguez-Reyes R., Capdevila C., Garcia-Mateo C., Caballero F.G. (2020). Positron Annihilation Spectroscopy Study of Carbon-Vacancy Interaction in Low-Temperature Bainite. Sci. Rep..

[51] Caballero F.G., Yen H.W., Miller M.K., Cornide J., Chang H.T., Garcia-Mateo C., Yang J.R. (2014). Three phase crystallography and solute distribution analysis during residual austenite decomposition in tempered nanocrystalline bainitic steels. Mater. Charact..

[52] Thuvander M., Weidow J., Angseryd J., Falk L.K.L., Liu F., Sonestedt M., Stiller K., Andrén H.O. (2011). Quantitative atom probe analysis of carbides. Ultramicroscopy.

[53] Meija J., Coplen T.B., Berglund M., Brand W.A., de Bièvre P., Gröning M., Holden N.E., Irrgeher J., Loss R.D., Walczyk T. (2016). Isotopic compositions of the elements 2013 (IUPAC Technical Report). Pure Appl. Chem..

